# Optic Disc Fundus Images Retain Biometric Identity Signals Under Deep Learning

**DOI:** 10.21203/rs.3.rs-8801056/v1

**Published:** 2026-06-05

**Authors:** Ali Azizi, Rafael Scherer, Aaron S. Rabinowitz, Douglas R. da Costa, Vitoria Palazoni Viegas Mendonça, Gustavo A. Samico, Gustavo R. Gameiro, Felipe A. Medeiros

**Affiliations:** University of Miami

**Keywords:** Biometric identification, Biometric data, Retinal imaging, Artificial Intelligence, Deep Learning, Convolutional Neural Networks

## Abstract

This work investigated whether deep learning models trained on optic disc-centered fundus images retain sufficient subject-specific information for biometric verification compared with models trained on full-field fundus photographs. A total of 30,836 color fundus photographs from 7,724 eyes of 4,500 subjects were obtained at the Bascom Palmer Eye Institute. Each fundus photograph was processed into three image representations: full-field fundus, optic disc region including 0.5 disc diameters of peripapillary retina, and tightly cropped optic disc only. Images were partitioned at the subject level into training (70%), validation (10%), and test (20%) sets. Separate Siamese convolutional neural network models were trained for each image type using triplet loss to learn subject-discriminative embeddings. Biometric verification was evaluated on the independent test set using exhaustive same-eye image pairing and cosine similarity. All image representations retained measurable subject-specific biometric signal. The full-fundus model achieved the highest performance (AUC, 0.992; EER, 4.4%), followed by the disc-region model (AUC, 0.989; EER, 5.5%) and the disc-only model (AUC, 0.969; EER, 10.5%). Accuracy was 0.968 for full fundus, 0.945 for disc region, and 0.919 for disc-only images. Pairwise comparisons showed significantly worse performance for disc-only images compared with full fundus (P < 0.001). Differences between full-fundus and disc-region models were small and not significant for AUC or EER. These findings demonstrate that deep learning models restricted to optic disc-centered fundus images retain meaningful subject-specific information, although performance declines as available retinal context is reduced. Inclusion of a narrow peripapillary rim yields biometric verification performance comparable to full-field fundus images. Although identity cannot be established from a fundus image without a linking key, recognizing that even restricted retinal regions retain subject-specific features highlights the importance of cautious data-sharing practices while supporting continued scientific collaboration.

## Introduction

Biometric identification systems increasingly rely on biological characteristics that are stable, distinctive, and difficult to replicate. Commonly used biometric modalities include fingerprints, facial features, iris patterns, and voice signatures, each offering different trade-offs in accuracy, robustness, and susceptibility to forgery [1]. Retinal fundus images represent a less commonly used but potentially powerful biometric modality, due to the uniqueness of retinal anatomy, particularly the vascular architecture, and its relative stability over time [2-4]. Unlike external biometric traits, the retina is anatomically shielded within the eye, making it less susceptible to environmental damage or intentional manipulation. These properties have long motivated interest in retinal imaging for biometric identification [1, 5].

Historically, retinal biometric systems have relied on feature extraction pipelines, often focused on segmentation and comparison of retinal vasculature patterns. However, with the advent of deep learning, there has been a shift toward representation learning approaches that automatically capture complex, high-dimensional features directly from images [6]. For example, convolutional neural networks (CNNs) have demonstrated strong performance across multiple biometric domains by learning subject-specific embeddings that enable accurate identity verification when a reference image is available [7]. In retinal imaging, most deep learning-based biometric studies have used full-field fundus photographs [1], implicitly assuming that wide-field retinal information is necessary to achieve reliable identification.

At the same time, cropped fundus images, particularly those centered on the optic disc, are widely used in ophthalmic research and artificial intelligence clinical applications [8, 9]. Optic disc-centered images are also commonly shared under the assumption that they are effectively de-identified [10], as they exclude large portions of the peripheral retina and are typically stripped of metadata and explicit patient identifiers. This assumption underlies many large-scale studies in glaucoma and other optic neuropathies, including deep learning frameworks that use optic disc images to predict structural and functional disease markers [11-13]. Despite their widespread use, however, the extent to which such cropped images retain subject-specific biometric information remains poorly understood. In fact, recent advances in deep learning raise important questions about these assumptions. Modern CNNs are capable of detecting subtle anatomical patterns that may not be visually apparent, including features related to vascular geometry, tissue reflectance, and structural relationships within localized retinal regions. It is therefore plausible that even restricted regions of the fundus, such as the optic nerve head and its immediate peripapillary surroundings, may still contain sufficient information to allow subject identification when analyzed using contemporary deep learning techniques. If so, this would have important implications for data-sharing practices, privacy considerations, and governance frameworks surrounding retinal imaging datasets.

To date, however, the specific contribution of the optic disc region to biometric identification has not been systematically evaluated at scale. Prior studies have either focused on full-field fundus images or relied on relatively small datasets [1], limiting their ability to assess identification performance under realistic clinical variability. Moreover, the relative performance trade-offs between full-field images and progressively restricted optic disc-centered crops remain unclear.

In this study, we evaluate whether deep learning models trained exclusively on optic disc-centered fundus images can achieve subject identification performance comparable to models trained on full-field fundus photographs. Using a large, heterogeneous clinical dataset, we compare biometric verification performance across three image representations: full-field fundus images, optic disc region images including a limited peripapillary rim, and tightly cropped optic disc-only images. By quantifying how identification performance changes as available retinal information is progressively constrained, we aim to clarify the extent to which cropped fundus images retain subject-specific biometric signals. These findings could inform both the technical understanding of retinal biometrics and the broader discussion surrounding de-identification assumptions and responsible data sharing in ophthalmic research.

## Methods

### Study Design, Ethical approval, and Data Source

This study utilized data obtained from the Advanced Imaging and Artificial Intelligence (AI)^2^ Laboratory at the Bascom Palmer Eye Institute (BPEI). Institutional Review Board approval was obtained, and the requirement for informed consent was waived. All procedures adhered to the ethical principles outlined in the Declaration of Helsinki. The dataset consisted of color fundus photographs from 4,500 patients, captured using non-mydriatic fundus cameras, including the Topcon TRC-NW400 (Topcon Corporation, Tokyo, Japan) and Canon CR-2 (Canon Inc., Ōta City, Tokyo, Japan). For inclusion, each eye was required to contribute at least two images of sufficient quality, based on internal assessment criteria that included clear visualization of the optic disc. Eyes with fewer than two qualified images were excluded.

### Image Preprocessing and Dataset Partitioning

**Figure 1:**
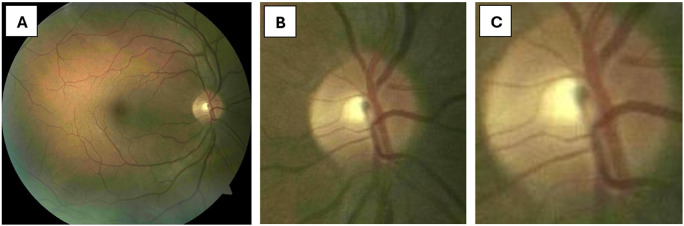
Image Preprocessing Pipelines for Fundus Photographs **(A)**Example of a full-field fundus image resized directly to 224 × 224 pixels without modification **(full fundus)**. Cropped versions of the same image are shown in **(B)** the optic disc + 0.5-disc-diameter region (**disc region**) and **(C)** the optic disc + 0.1-disc-diameter region (**disc only**). The optic disc was localized using a YOLOv5 object-detection model. A square region centered on the detected disc was extracted only if the model’s confidence was ≥ 0.9 and at least 0.5 disc or 0.1 disc-diameter of surrounding retina was visible in all directions for the disc-region and disc-only versions, respectively. All cropped regions were subsequently resized to 224 × 224 pixels for downstream analysis.

Each image underwent three distinct preprocessing pipelines. In the first, the original full-field fundus image (referred to as the full fundus) was directly resized to 224 × 224 pixels. In the second, the optic disc region was localized using a YOLO-v5 (You Only Look Once, version 5) object detection model (referred to as the disc region). A square crop centered on the optic disc was retained only if the detection confidence was ≥ 0.9 and at least 0.5 disc diameters of the surrounding retina were visible in all directions. In the third pipeline, the cropping procedure was identical to the second, except that the retained region included only 0.1 disc diameters of surrounding retina (referred to as the disc only). The resulting disc-only images contained minimal peripapillary context, primarily accounting for localization margin and ensuring robust optic disc detection across all images. These cropped images were also resized to 224 × 224 pixels for downstream analysis (Fig. 1). All images (full fundus, disc region, and disc only) were partitioned at the patient level into training (70%), validation (10%), and test (20%) subsets, ensuring that no subject contributed images to more than one set.

### Deep Learning Model Architecture, Training, and Optimization

**Figure 2:**
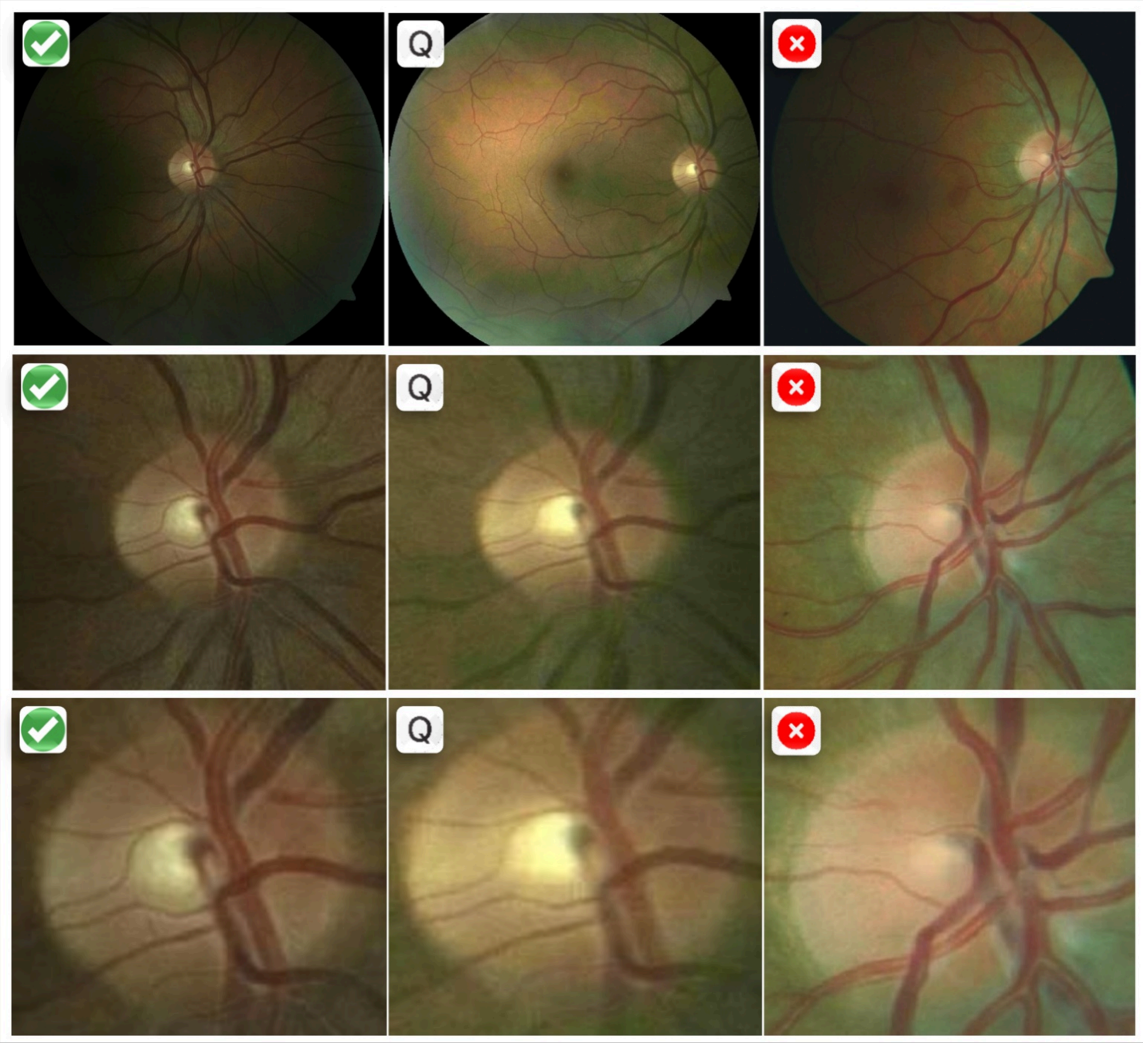
Representative Samples Used in Siamese Triplet Neural Network Training Examples of full-field fundus images (**full fundus**; top row) and their cropped versions: optic disc + 0.5-disc-diameter region (**disc region**; middle row) and optic disc + 0.1-disc-diameter region (**disc only**; bottom row). Each triplet set consists of an anchor (**Q**), a positive sample from the same subject (**green check mark**), and a negative sample from a different subject (**red X**). Triplet sets were generated during training to guide the network in learning feature embeddings that cluster images from the same subject while separating those from different subjects.

Biometric identification was performed using a Siamese triplet neural network implemented in PyTorch. Several convolutional backbones were explored, including ResNet-18, ResNet-50, EfficientNet-B0, MobileNet-V3, VGG-16, and RETFound. The final configuration employed RETFound for the full-fundus images and EfficientNet-B0 for the disc-region and disc-only images. Triplet samples, each comprising an anchor, a positive (same subject), and a negative (different subject) image, were dynamically generated during training (Fig. 2). To learn discriminative embeddings, we used a margin-based triplet loss function structured to bring images from the same subject closer together while separating those from different subjects. Each triplet ($x_{a}$, $x_{p}$, $x_{n}$ consists of an anchor image ($x_{a}$), a positive image ($x_{p}$), and a negative image ($x_{n}$). The model learns L2-normalized embeddings f(x), and optimizes the following objective:

$$L_{triplet}=max\;(0,\;D\;(f\;(x_{a})\;,\;f\;(x_{p}))-D\;(f\;(x_{a})\;,\;f\;(x_{n}))+\alpha \;)$$

where D(⋅,⋅) represents cosine distance between embeddings, and α is a margin parameter (set to 1.0). This loss encourages embeddings from the same individual to be closer than those from different individuals by at least the margin α, thereby enhancing the model’s discriminative power. To facilitate hard-negative mining, cosine-distance embeddings were first computed for all training and validation images; for each anchor, we selected the 100 most similar images to enable preferential selection of visually challenging positives and negatives. All embeddings were L2-normalized to unit length, allowing cosine similarity to serve as the distance metric throughout training and inference. Model training utilized the Adam optimizer with an initial learning rate of 1 × 10^−4^ and a batch size of 16. Early stopping was applied with a patience of five epochs, and a learning rate scheduler was used with a patience of three epochs. The training loop also computed retrieval metrics such as Recall@K and Mean Reciprocal Rank (MRR), which were logged using the Weights & Biases (wandb) platform. Hyperparameter tuning, including learning rate, margin (α), embedding dimensionality, neck usage, and backbone selection, was conducted using Bayesian optimization based on the validation loss. The best-performing model, as determined by the lowest validation triplet loss, was retained for final evaluation.

### Embedding Inference and Pairwise Evaluation

The primary objective of this study was to determine whether restricting the input of a deep learning model to the optic disc region of fundus images preserves sufficient subject-specific information for reliable biometric identification.

After model training, biometric identification was evaluated on a held-out test set independent of the training and validation data. Verification was framed as a pairwise comparison task, in which all possible pairs of fundus images within the test set were generated and classified as belonging to the same subject or to different subjects. To avoid confounding effects related to anatomical differences between eyes, only pairs from the same eye (right-right or left-left) were included, and cross-eye comparisons were excluded.

**Figure 3:**
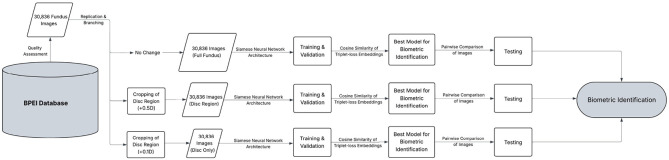
Workflow for Biometric Identification Using Full-Field and Cropped Optic Disc-Region Images. This schematic illustrates the study pipeline for evaluating the efficacy of full-field versus optic disc–focused fundus images in biometric identification. A total of 30,836 fundus images from the Bascom Palmer Eye Institute (BPEI) dataset were subjected to quality assessment, including verification of clear optic disc visualization, and were replicated twice for parallel processing. The first branch retained full-field images (**full fundus**), while the other two used a pre-trained deep learning model to crop the optic disc region at two scales: **disc region** (+0.5-disc diameter) and **disc only**(+0.1-disc diameter). Each image set (full fundus, disc region, and disc only) was then processed through Siamese neural networks with triplet-loss training and validation strategies. Cosine similarity of the resulting embeddings was used to identify the best-performing model for each image type. These models were subsequently applied to pairwise biometric identification tasks, determining whether two input images originated from the same subject.

For each image pair, both images were independently passed through the trained Siamese network to produce fixed-length feature embeddings. Similarity between embeddings was quantified using cosine similarity, with higher values indicating greater likelihood that the images originated from the same individual. This embedding-based verification framework enables assessment of identity information without explicit classification and is consistent with standard biometric evaluation approaches. Figure 3 provides an overview of the workflow from image preprocessing and embedding generation to pairwise comparison.

### Statistical Analysis

Verification performance was assessed on the held-out test set for each image representation (full fundus, disc region, and disc only) at the level of pairwise image comparisons. Similarity scores were converted into binary match or non-match decisions using operating thresholds derived from the validation set. Two thresholding strategies were used: the equal-error rate (EER), defined as the point at which false-accept and false-reject rates are equal, and the Youden index, which maximizes the sum of sensitivity and specificity.

Receiver operating characteristic (ROC) and detection error tradeoff (DET) curves were generated for each model. Reported performance metrics included area under the ROC curve (AUC), EER, genuine-accept rate (GAR), false-accept rate (FAR), false-reject rate (FRR), accuracy, sensitivity, and specificity (the latter three evaluated at the Youden index operating point). Bootstrap percentile 95% confidence intervals (95% CIs) for AUC and EER were estimated using 1,000 resampling iterations.

Pairwise comparisons between models were performed using McNemar’s test on aligned binary decisions obtained at the fixed EER and Youden thresholds, enabling direct comparison of classification outcomes on identical image pairs. A two-tailed p-value < 0.05 was considered statistically significant. All analyses were conducted using Python version 3.11.

## Results

A total of 30,836 color fundus images from 7,724 eyes of 4,500 subjects that met the quality inclusion criteria were used to investigate retinal biometric identification. The identification performance of the full-fundus model was compared with that of the disc-region and disc-only models.

All three models demonstrated strong ability to distinguish whether paired fundus images originated from the same subject, indicating that subject-specific biometric signal was preserved across all image representations. Overall identification performance, however, varied systematically with the amount of retinal information available. The model trained on full-field fundus images achieved the highest performance, with an accuracy of 0.968 on the held-out test set, significantly outperforming both the disc-region model (accuracy 0.945) and the disc-only model (accuracy 0.919) (all p < 0.001).

Pairwise comparisons using McNemar’s test on aligned image pairs provided further insight into these differences. The full-fundus model demonstrated significantly higher accuracy, sensitivity, and specificity than the disc-only model (all p < 0.001). When compared with the disc-region model, the full-fundus model showed a smaller but statistically significant advantage in accuracy and specificity (both p < 0.001), whereas sensitivity did not differ significantly between the two models (p = 0.406). These findings indicate that inclusion of a narrow peripapillary rim around the optic disc substantially mitigates the performance loss observed when using disc-only images. Detailed quantitative performance metrics for all three models are summarized in Table 1.

## Discussion

In this study, we evaluated how progressively restricting retinal input to the optic disc region affects deep learning-based biometric identification. Using Siamese convolutional neural networks trained on full-field fundus images, optic disc-centered images with limited peripapillary context, and tightly cropped optic disc-only images, we observed a graded decline in identification performance as available retinal information decreased. Models trained on full-field fundus images achieved the highest overall performance, whereas models restricted to the optic disc alone performed worse but still retained meaningful subject-specific signal. Notably, inclusion of a narrow peripapillary rim around the optic disc yielded identification performance comparable to full-field images, indicating that this region contains a substantial proportion of the biometric information present in the fundus. Our analysis focused on pairwise verification, i.e., determining whether two images belonged to the same individual, and should not be taken to imply that identity can be inferred from a single fundus image in isolation without a corresponding reference image.

Biometric system performance is commonly evaluated using metrics that balance security and usability, particularly the EER, which represents the operating point at which false-accept and false-reject rates are equal. As a pragmatic benchmark, EERs below 1% are often considered very high performance, values between 1% and 5% are typical of competitive commercial systems, and values exceeding 10% are generally regarded as insufficient for high-assurance applications, although thresholds vary by modality and protocol [14, 15]. In this context, our full-fundus model achieved an EER of 4.4% (AUC = 0.992), which was not statistically different from the disc-region model’s EER of 5.5% (AUC = 0.989). In contrast, the disc-only model exhibited a substantially higher EER of 10.5% (AUC = 0.969). While reduced performance with constrained input is expected, the observation that even tightly cropped optic disc images retain identifiable characteristics detectable by deep learning warrants careful consideration.

Previous work in retinal biometrics has largely relied on handcrafted feature extraction pipelines, most commonly focusing on retinal vascular patterns across the full fundus. These approaches have reported high recognition rates in small, publicly available datasets that are predominantly composed of healthy subjects [1, 3, 16-20]. In contrast, our study evaluated a substantially larger and more heterogeneous clinical dataset comprising 4,500 subjects and more than 30,000 fundus images. Despite this increased scale and variability, identification accuracies for the full-fundus model (0.97) were consistent with previously reported values using full-field images [21-23], while disc-region and disc-only models achieved accuracies of 0.95 and 0.92, respectively. These findings extend prior work by demonstrating that meaningful subject-specific signal persists even when deep learning models are restricted to a small fraction of the retinal image, particularly within the optic nerve head and adjacent peripapillary vasculature [24, 25].

From a practical perspective, the observation that optic disc-centered images with limited peripapillary context achieve identification performance comparable to full-field fundus images has several technical implications. Disc-centered imaging is increasingly common in both clinical care and research, driven by widespread availability of non-mydriatic cameras and automated image alignment [26-28]. Restricting analysis to the optic disc region can substantially reduce computational complexity, storage requirements, and bandwidth demands while preserving strong identification performance. Concentrating on this anatomically information-dense region also functions as an implicit feature selection step and may reduce reliance on peripheral image content or dataset-specific background artifacts. Nevertheless, the highest identification performance was consistently achieved when full-field retinal information was available, underscoring the additional discriminative value contributed by broader retinal context.

These findings also inform ongoing discussions about whether retinal images should be considered biometric data. Under current U.S. privacy regulations, retinal images are not explicitly classified as biometric identifiers, and professional organizations such as the American Academy of Ophthalmology have emphasized that the likelihood of re-identification from de-identified retinal images is extremely low [29]. Crucially, although our results demonstrate that deep learning models can extract subject-specific signal from cropped fundus images, meaningful identification remains fundamentally dependent on the existence of a previously acquired reference image. In the absence of such a reference, or a linked identity database, the probability of re-identification from an isolated retinal image remains very low in practical terms [30]. Thus, our findings do not imply that cropped or de-identified fundus images enable standalone identification, but rather that they retain latent identity-related information that could be leveraged only under specific, constrained conditions.

At the same time, as artificial intelligence methods continue to advance, assumptions about de-identification based solely on image cropping may warrant periodic re-evaluation. This does not diminish the substantial public health benefits of sharing retinal imaging data for research and AI development, but instead underscores the importance of proportionate data governance strategies, including data-use agreements, access controls, and emerging technical safeguards that prevent misuse while enabling continued scientific collaboration.

Several technical strategies may help reconcile privacy protection with scientific utility in retinal imaging. Recent work has demonstrated that identity-related information in fundus images can be selectively attenuated through latent-space manipulation while preserving clinically relevant features, such as disease-related structures and lesions [31]. When combined with complementary safeguards, such as on-device processing, controlled-access environments, and secure computation frameworks, these approaches could enable the sharing of retinal images that retain diagnostic and research value while substantially reducing re-identification risk. Continued research focused on disentangling identity-related and disease-related representations within deep learning models may further advance privacy-preserving retinal imaging without compromising medical utility.

This study has several limitations. First, we did not assess the longitudinal stability of biometric identification in eyes undergoing substantial pathological change, which may affect the persistence of subject-specific features over time. Second, we did not evaluate cross-eye identification between left and right eyes, an important consideration given potential similarities arising from shared genetic or systemic factors. Third, our evaluation was framed as a verification task rather than explicit one-to-many identification, although the embedding-based framework used here is readily extensible to open-set identification scenarios. Finally, we did not examine the relative biometric contribution of retinal regions outside the optic disc. Despite these limitations, the large scale and clinical heterogeneity of the dataset strengthen the generalizability of our findings to real-world ophthalmic imaging settings.

In conclusion, deep learning models restricted to the optic disc region retain meaningful subject-specific information and can achieve substantial biometric identification performance, although performance degrades as available retinal input is reduced. Inclusion of limited peripapillary context yields performance comparable to full-field fundus images, underscoring the richness of the optic nerve head region. These findings have important implications for the interpretation of de-identification practices and support the continued development of responsible data-sharing strategies that balance privacy protection with the advancement of ophthalmic research and artificial intelligence.

## Figures and Tables

**Figure 4:**
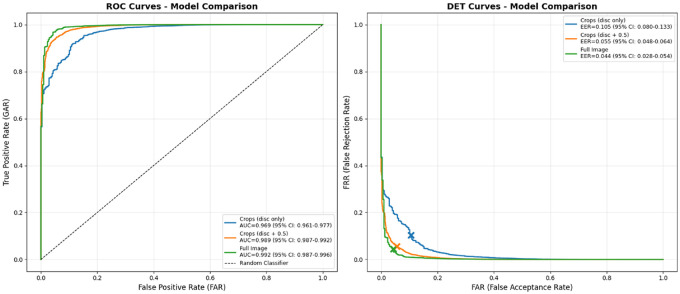
Receiver Operating Characteristic (ROC) and Detection Error Tradeoff (DET) Curves for biometric identification models. ROC curves (left) and DET curves (right) compare verification performance among the three Siamese neural network models trained on full-fundus, disc-region (+0.5 D), and disc-only (+0.1 D) fundus images. The ROC curves plot the genuine-accept rate (GAR; equivalent to true-positive rate) against the false-accept rate (FAR; equivalent to false-positive rate), with the area under the curve (AUC) summarizing overall discrimination performance. The DET curves illustrate the tradeoff between the false-accept rate (FAR) and false-reject rate (FRR) across thresholds; the equal-error rate (EER) indicates the point where FAR = FRR. The full-fundus model achieved the best performance (AUC = 0.992 [95% CI, 0.987–0.996]; EER = 0.044 [0.028–0.054]), followed by the disc-region (AUC = 0.989; EER = 0.055) and disc-only (AUC = 0.969; EER = 0.105) models.

**Table 1:** Biometric Identification Performance of Siamese Neural Network Models

Performance Metric [95% CI]	Full Fundus	Disc Region (+ 0.5D)	Disc Only (+ 0.1D)
**Accuracy**	0.968 [0.967, 0.969]	0.945 [0.944, 0.946] †	0.919 [0.918, 0.920] †
**Sensitivity**	0.957 [0.928, 0.975]	0.946 [0.928, 0.960]	0.888 [0.845, 0.920] †
**Specificity**	0.968 [0.967, 0.969]	0.945 [0.944, 0.946] †	0.919 [0.918, 0.920] †
**AUC**	0.992 [0.987, 0.996]	0.989 [0.987, 0.992]	0.969 [0.961, 0.977] †
**EER**	0.044 [0.028, 0.054]	0.055 [0.048, 0.064]	0.105 [0.080, 0.133] †
**FAR**	0.032 [0.031, 0.033]	0.055 [0.054, 0.056] †	0.081 [0.080, 0.083] †
**FRR**	0.043 [0.025, 0.072]	0.054 [0.040, 0.072]	0.112 [0.080, 0.155] †

**Note 1:** Accuracy, sensitivity, and specificity were reported at the Youden thresholds of 0.62, 0.57, and 0.65 for full-fundus, disc-region, and disc-only models, respectively.

Note 2:

† indicates a statistically significant p-value (< 0.001) in comparison with the full-fundus image model, as determined by McNemar’s test.

**Abbreviations**: AUC = area under the ROC curve; EER = equal-error rate; FAR = false-accept rate; FRR = false-reject rate.

ROC and DET curves for the three models are shown in Fig. 4. The full-fundus model showed significantly superior discrimination compared with the disc-only model, with higher AUC (0.992 vs 0.969), lower equal-error rate (4.4% vs 10.5%), and lower false-accept and false-reject rates (all p < 0.001). In contrast, no statistically significant differences were observed between the full-fundus and disc-region models for AUC (0.992 vs 0.989; p = 0.246), equal-error rate (4.4% vs 5.5%; p = 0.160), or false-reject rate (p = 0.401), although the full-fundus model maintained a significantly lower false-accept rate (p < 0.001).

## Data Availability

The data supporting the findings of this study were obtained from the Bascom Palmer Eye Institute, University of Miami and are not publicly available due to restrictions related to participant confidentiality and institutional/ethical approvals. The code used for the deep learning model and analyses is available from the corresponding author upon reasonable request.
